# A Split Face Study Comparing the Effect of a PDLLA Based Product and PLLA on the Nasolabial Fold (NLF)

**DOI:** 10.1111/srt.70324

**Published:** 2026-01-14

**Authors:** Je‐Young Park, Dong Jin Im, Hee Dae Jeon, Hyun Jo Kim, Do Young Rhee, BonCheol Leo Goo, Kwang Ho Han, Zhongfan Chang, Hei Sung Kim

**Affiliations:** ^1^ Apgujeong Oracle Dermatology Clinic Seoul South Korea; ^2^ Lisienne Dermatology Clinic Gyeonggi‐do South Korea; ^3^ Banobagi Dermatologic Clinic Seoul South Korea; ^4^ CNP Skin Clinic Cheonan South Korea; ^5^ Heal House Dermatology Clinic Seoul South Korea; ^6^ Naeum Dermatology and Aesthetics Clinic Seoul South Korea; ^7^ Nature Dermatology Clinic Seoul South Korea; ^8^ Department of Dermatology Asan Medical Center Seoul South Korea; ^9^ Department of Dermatology Incheon St. Mary's Hospital College of Medicine The Catholic University of Korea Seoul South Korea

**Keywords:** hyaluronic acid, L‐lactic acid, PDLLA based product, poly‐D, poly‐L‐lactic acid, nasolabial fold

## Abstract

**Background:**

Injectable Poly‐L‐lactic acid (PLLA) is effective in restoring mid‐face volume and is widely used treating the nasolabial fold (NLF). This study aimed to compare the safety and efficacy of a novel PDLLA based product (PDLLA + non‐cross‐linked HA; Poly‐D, L‐lactic acid + hyaluronic acid) with that of a commonly used PLLA for NLF correction.

**Methods:**

In this multi‐center, randomized, split‐face, evaluator‐blinded study, 33 subjects received injections of the PDLLA based product on one NLF and PLLA injection on the other (three injections total, administered at 4‐week intervals). Wrinkle severity was assessed using standardized photographs taken at baseline (week 0); before the second and third injections (weeks 4 and 8); 4 weeks after the third injection (week 12); and 3 months after the final injection (week 24) using the Wrinkle Severity Rating Scale (WSRS). Safety data were collected through subject interviews and case report forms.

**Results:**

Both the PDLLA based product and PLLA significantly improved NLF wrinkle severity at all time points compared to baseline (*p* < 0.001). The degree of improvement was comparable between the two products throughout the observation period.

**Conclusion:**

The PDLLA based product demonstrated non‐inferiority to PLLA for NLF correction. Further studies are needed to assess long‐term safety.

## 1 Introduction

The nasolabial folds (NLFs) refer to a pair of skin creases which extend from each side of the nose to the corners of the mouth. Over the course of aging, the creases grow in both length and depth becoming more prominent and permanent [1].

Injectable fillers [2] have become extremely popular for soft tissue contouring and volumizing and are often applied to the NLF [3, 4, 5]. Poly‐L‐lactic acid (PLLA) is a well‐established biostimulator [6] that promotes collagen synthesis and provides gradual, long‐lasting volumization, particularly in the mid‐face and NLF [7, 8, 9, 10]. However, its relatively slow onset of effect and potential for nodule formation remain limitations.

Injectable Poly‐D, L‐lactic acid (PDLLA) + non‐crosslinked hyaluronic acid (HA) is a new PDLLA based product. Similar to PLLA, PDLLA is biodegradable and biocompatible, and promotes collagen synthesis [11, 12]. However, PDLLA is composed of spongiform microspheres with a smooth outer shape and an inner reticular, porous structure, while PLLA comprises irregularly shaped spiky particles with a densely packed inner structure [13, 14, 15]. The amorphous structure of PDLLA allows it break down relatively quickly. Since PDLLA disintegrates from the core, there is gradual release of acidic degradation products, allowing it to have good tissue biocompatibility [16]. Furthermore, its combination with non‐crosslinked HA offers immediate hydration and plumpness, while moderating the tendency of nodule formation within the skin [17, 18].

Given the structural and compositional differences between PDLLA and PLLA, direct comparative data are needed to assess whether a PDLLA‐based product provides similar or superior efficacy and safety in clinical practice. Our split‐face study allows for controlled, within‐subject comparison to eliminate inter‐individual variability, thus enhancing the clinical relevance of the findings for aesthetic practitioners seeking alternatives to PLLA.

## 2 Materials and Methods

### 2.1 Study Design and Participants

**FIGURE 1:**
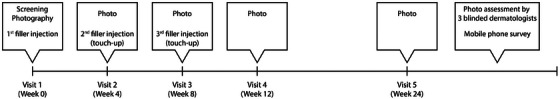
Study layout (Scheduled visits and assessment time‐points).

This study was a multi‐center (four dermatology centers were involved), evaluator‐blinded, randomized, split‐face controlled trial. At visit 1 (week 0), subjects were screened for NLF severity, and eligible subjects gave their written informed consent to participate in the study. The treatment consisted of a total of three injections (week 0, 4, and 8) where subjects received a PDLLA based product on one side and PLLA injection on the other NLF. Follow‐up visits were made 1‐ and 3‐months after the third injection (Figure 1).

We enrolled healthy adults aged 30–70 years who desired improvement of their NLFs, as determined by a pretreatment Wrinkle Severity Rating Scale (WSRS) score of ≥2 on both folds (1‐ “NLF absent”, 2‐ “mild NLF”, 3‐ “moderate NLF”, 4‐ “severe NLF”, 5‐ “extreme NLF”). Participants were excluded if they met any of the following conditions: (1) pregnant or nursing, or planning pregnancy during the study period; (2) presence of severe, progressive, or clinically significant medical conditions that could interfere with study outcomes; (3) known hypersensitivity or history of allergic reactions to PDLLA, HA, PLLA, or lidocaine; (4) prior facial tissue augmentation with temporary, semipermanent, permanent fillers, or threads; (5) tendency to form keloids or hypertrophic scars; or (6) facial hair that could interfere with standardized visual assessments.

The study was conducted in accordance with good clinical practice, conforming to the ethical principles of the Declaration of Helsinki and approved by the Institutional Review Board (IRB) of Incheon St. Mary's Hospital, The Catholic University of Korea (IRB number: OC24RASI0073). Consent for study participation as well as publication of identifiable images was obtained.

### 2.2 Method of Administration

Both the PDLLA based product (PDLLA 170 mg + non‐crosslinked HA 30 mg, Juvelook Volume, VAIM Co. Ltd, Okcheon, Korea) and PLLA (Sculptra, Dermik Laboratories, Bridgewater, NJ, USA) are supplied as vials of lyophilized powder. Both were reconstituted in 6 mL of sterile water and left to stand for 24 h. Prior injection, the vials were vortexed for 30 min and then 2% lidocaine was added by a two‐way syringe, making a final 8 mL solution [19, 20, 21, 22].

The procedure was performed with the patient under local anesthesia. A layer of anesthetic cream (EMLA, lidocaine 2.5% and prilocaine 2.5%, AstraZeneca AB, Sodertalje, Sweden) was applied over the treatment area for 30 min. First, a puncture was made on the entry point with a 23G needle followed by filler injection with a 60 mm, 23G blunt tip microcannula. The two different filler products were randomly delivered either to the right or left NLF targeting the deep fat and supra‐periosteal layer. A volume of 2 mL/side was applied in the first session, with a touch‐up of up to 2 mL/side (average of 1 mL/side) in the following two sessions.

### 2.3 Clinical Assessment

Three‐dimensional (3D) images were taken before the procedures (week 0, week 4, week 8), and at all follow‐up visits (week 12, week 24) with a facial skin analyzer (3D Meta‐Vu, PSI Plus Co., LTD, Suwon, Korea). Treatment efficacy was based on the WSRS score change and the 5‐point Global Aesthetic Improvement Scale (GAIS) (3‐ “very much improved”, 2‐ “much improved”, 1‐ “improved”, 0‐ “no change”, −1‐ “worse”) scoring rated by three independent, blinded, board‐certified dermatologists. After the final visit, a mobile phone survey was sent out to the study participants who were asked to rate their overall satisfaction using the following scale: “greatly satisfied”, “satisfied”, “slightly satisfied”, or “not satisfied”. The safety and tolerability of the study products were assessed from AE data collected at each visit.

### 2.4 Statistical Analysis

In our analysis, the primary aim was to evaluate the outcomes of ordinal variables (WSRS and GAIS scores) [23, 24] at all time‐points during the study period. For this, we computed the mean values with 95% confidence intervals (CIs) for these variables. To quantify the magnitude of change, we determined the mean differences by comparing the values against the baseline value.

To demonstrate that the PDLLA based product is not inferior to the existing PLLA treatment, we performed a non‐inferiority test between the two fillers. We assumed no difference between the PDLLA based product and PLLA in terms of WSRS score change/GAIS score and calculated non‐inferiority using a 15% margin of non‐inferiority at a one‐sided significance level of 0.05.

All statistical analyses conducted were performed using the SPSS (Ver.25, IBM corporation, Armonk, NY, USA) with statistical significance wet at *p* < 0.05.

## 3 Results

A total of 33 individuals aged between 33 and 66 years (mean age of 48.5 ± 11.6 years; 24 (72.7%) female) were included in the study (Table 1). According to the WSRS assessment conducted by three blinded independent evaluators, the participants initially had a mean WSRS score of 3.39 (95% CI 3.27–3.53) for their NLF.

**TABLE 1:** Clinical characteristics of the subjects at baseline.

Characteristics	*N* = 33
Age, years, mean (SD)	48.5 (11.6)
*Age, years, n (%)*	
<40	14 (42.4)
40–49	5 (15.2)
50–59	5 (15.2)
≥60	9 (27.3)
*Sex, n (%)*	
Male	9 (27.3)
Female	24 (72.7)
*WSRS, mean (95% CI)*	
Initial (pre‐treatment)	3.39 (3.27–3.53)

All 33 subjects completed three sessions of treatment with the average amount of administered by the end of the two follow‐up sessions being 4 mL/side (2 mL/side applied on the first session, with an average of 1 mL/side applied in the following two sessions).

**FIGURE 2:**
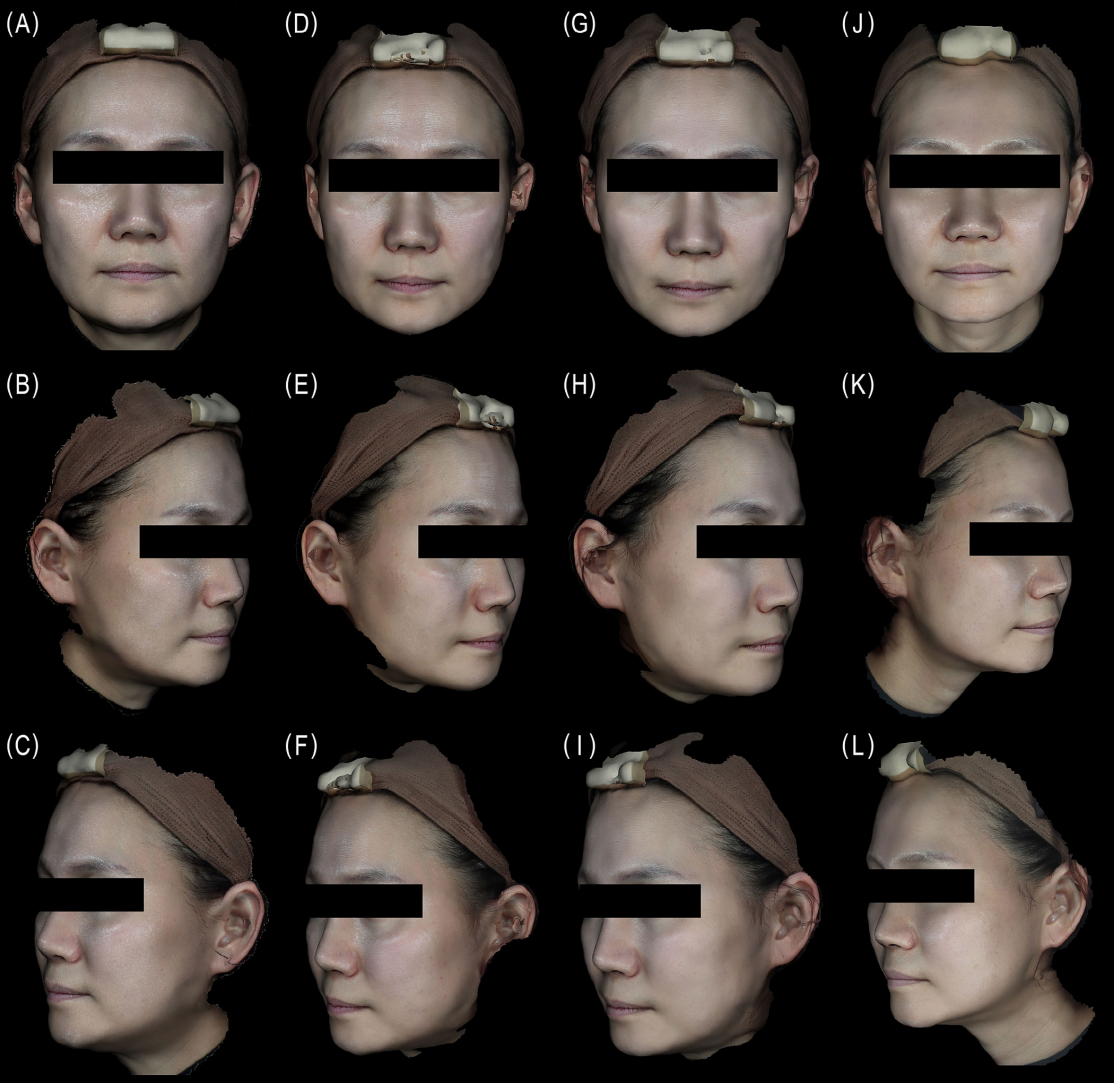
3D photos of a 48‐year‐old female at baseline (A, B, C) week 4 (D, E, F), week 8, (G, H, I), and week 24 (J, K, L). The WSGS score of the NLF changed from a 3 (“moderate NLF”) to a 1 (“NLF absent”) on both sides at week 24 (right: Sculptra, left: Juvelook Volume).

**FIGURE 3:**
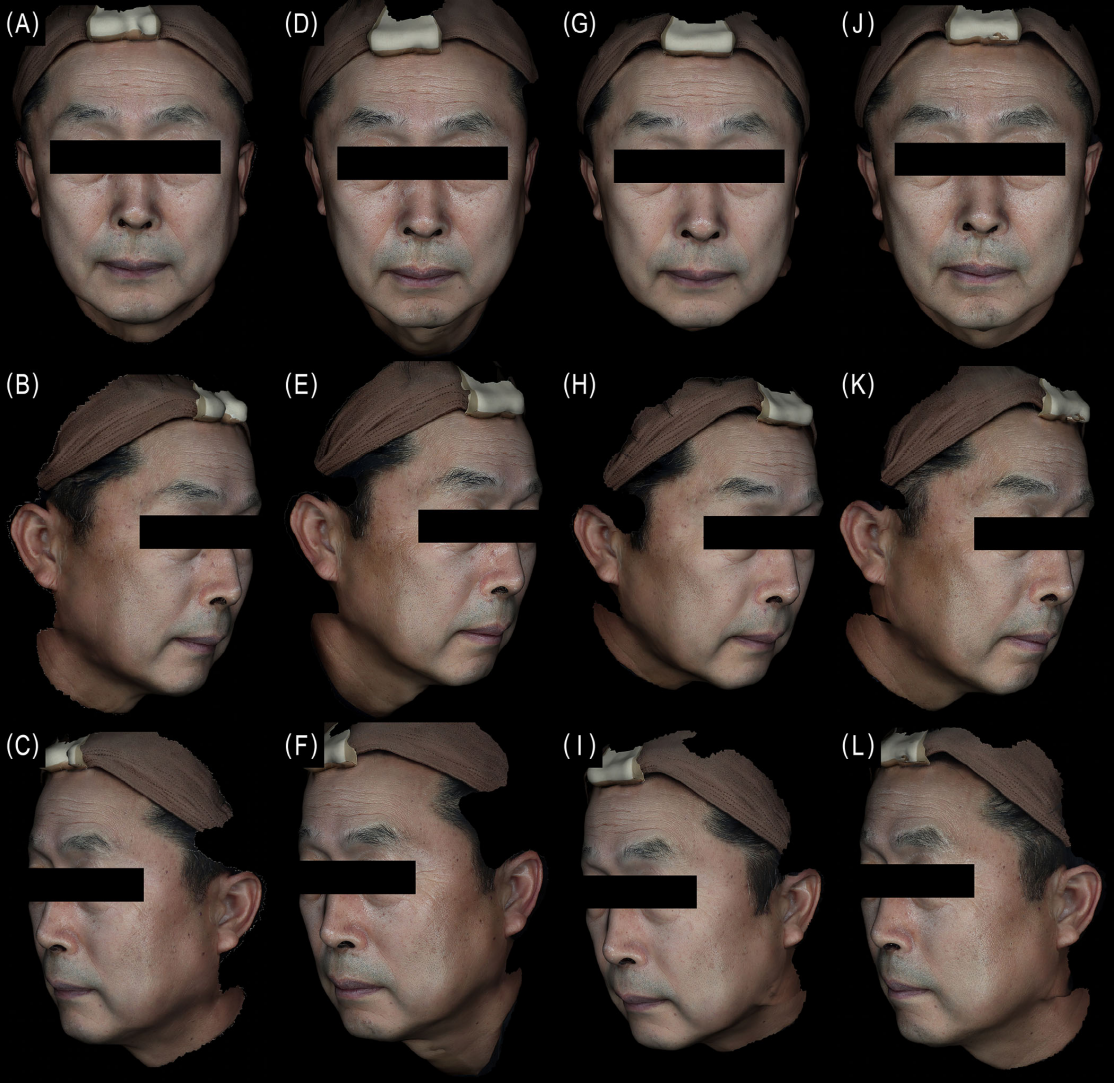
3D photos of a 69‐year‐old male at baseline (A, B, C) week 4 (D, E, F), week 8, (G, H, I), and week 24 (J, K, L).The WSGS score of the NLF changed from a 4 (“severe NLF”) to a 2 (“mild NLF”) on both sides at week 24 (right: Sculptra, left: Juvelook Volume).

**FIGURE 4:**
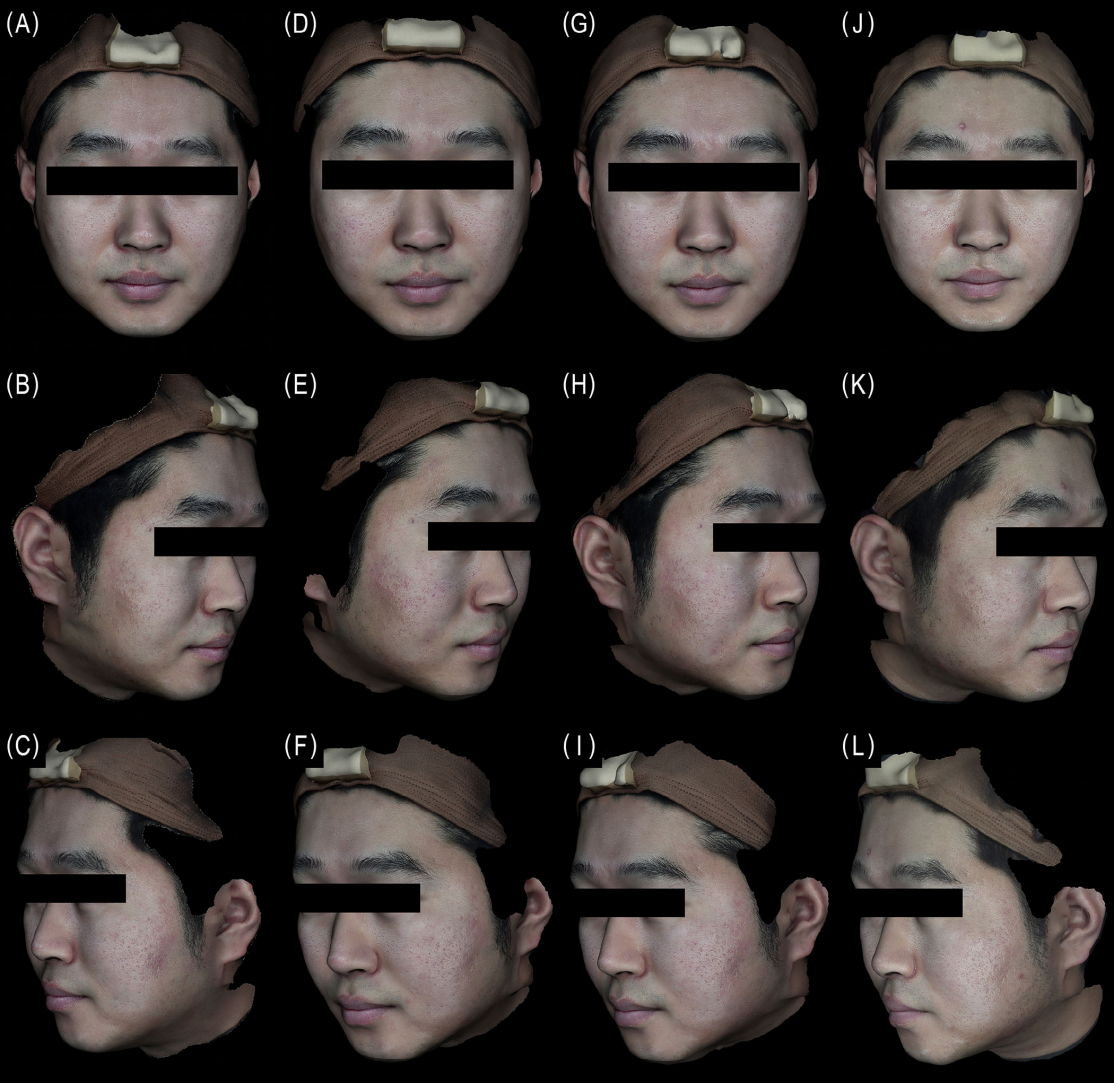
3D photos of a 35‐year‐old male at baseline (A, B, C) w4 (D, E, F), week 8, (G, H, I), and week 24 (J, K, L). The WSGS score of the NLF changed from a 3 (“moderate NLF”) to 2 (“mild NLF”) on both sides at week 24 (right: Juvelook Volume, left: Sculptra).

A significant decrease in the mean WSRS score was observed following the PDLLA based product injection, beginning at week 4 (mean difference −0.81; 95% CI −0.95 to −0.66) and continuing up to week 24 (*p* < 0.001, at all time‐points vs. baseline) (Table 2). The mean WSRS score reduction was also significant on the PLLA injected side from week 4 to week 24 (*p* < 0.001, at all time‐points vs. baseline) (Table 2). Furthermore, WSRS score reduction from the PDLLA based product was comparable to that of PLLA with *p*_non‐inferiority_ being > 0.05 (Figures 2, 3, 4). We also found no significant difference between the PDLLA based product and injectable PLLA in terms of GAIS at weeks 4, 8, 12, and 24 (Table 2).

**TABLE 2:** Primary outcome (WSRS score change, GAIS score) between Juvelook volume (injectable PDLLA + non cross‐linked HA) and Sculptra (injectable PLLA).

	Week 0 (Baseline)	Week 4 (Before second injection)	Week 8 (Before third injection)	Week 12 (At 4‐week FU after Tx)	Week 24 (At 12‐week FU after Tx)
***Injection with Juvelook volume***					
WSRS score Mean (95% CI)	3.42 (3.30–3.55)	2.62 (2.42–2.81)	2.16 (1.99–2.34)	1.99 (1.82–2.16)	2.05 (1.87–2.23)
WSRS score change Mean (95% CI)	Reference	**−0.81 (−0.95 to −0.66)**	**−1.26 (−1.40 to −1.12)**	**−1.43 (−1.58 to −1.29)**	**−1.37 (−1.54 to −1.21)**
GAIS score Mean (95% CI)	Reference	**1.07 (0.90–1.24)**	**1.61 (1.42–1.79)**	**1.84 (1.64–2.03)**	**1.85 (1.64–2.06)**
***Injection with Sculptra***					
WSRS score Mean (95% CI)	3.36 (3.23–3.50)	2.58 (2.38–2.77)	2.29 (2.10–2.48)	2.03 (1.85–2.21)	2.16 (1.87–2.35)
WSRS score change Mean (95% CI)	Reference	**−0.79 (−0.94 to −0.64)**	**−1.07 (−1.22 to −0.92)**	**−1.33 (−1.47 to −1.19)**	**−1.20 (−1.37 to −1.04)**
GAIS score Mean (95% CI)	Reference	**1.09 (0.90–1.29)**	**1.42 (1.22–1.63)**	**1.74 (1.53–1.94)**	**1.62 (1.40–1.83)**
***p value for the comparison of the primary outcome between the two groups (non‐inferiority test)***					
WRSR score change	—	>0.05	> 0.05	>0.05	>0.05
GAIS score	—	>0.05	>0.05	>0.05	>0.05

Twenty subjects responded to the mobile phone survey where 40% were “greatly satisfied”, 40% “satisfied”, and 20% “slightly satisfied” with the overall outcome. Although 70% of the respondents had no preference between the two products, 15% preferred the PDLLA based product over PLLA injection whereas 15% preferred the PLLA. Overall, noninferiority of the PDLLA based product versus injectable PLLA was successfully demonstrated over the 24‐week study period.

In terms of injection site reaction, most replied to have experienced mild swelling for 3–4 days regardless of the product with few reporting the initial swelling to be slightly more prominent with the PDLLA based product. One particular patient developed moderate swelling and tenderness on the PDLLA based product treated side following the second injection, which quickly resolved after intralesional triamcinolone (2.5 mg/mL) and hyaluronidase injection to the site.

## 4 Discussion

With the population's exceptional desire to look young and easy access to noninvasive cosmetic therapy, Korea pioneers in innovative filler products [17, 25, 26]. That being said, the market share of biostimulators has doubled within a recent span of 3–4 years. This trend signifies an increasing acceptance and demand for biostimulators in the Korean market.

PDLLA injection is the newest era for bio‐stimulation. Among the PDLLA products, Juvelook Volume has received worldwide attention with its unique properties. The aim of this randomized, split‐face comparison was to provide data on the effectiveness and safety of this new PDLLA based product on the NFL and to show its noninferiority to the industry standard Sculptra (PLLA).

All subjects in this study were Korean with most being women. There was a higher proportion of individuals in their 30s than expected which reflects the Gen Z trend of prioritizing early intervention to the natural signs of aging. Owing to the fact that a proportion of women in their 40s and 50s (who are the ideal candidate with a prominent NLFs and the best spending power) already received filler injection or a thread lift prior enrollment (and were excluded from the study), they comprised a smaller share of the total study population.

The primary effectiveness variable in this study was the WSRS score change and GAIS score which showed NFL improvement at all visitation time‐points (week 4, week 8, week 12, and week 24) versus baseline with both the PDLLA based product and injectable PLLA. In addition, noninferiority of the PDLLA based product versus injectable PLLA was successfully demonstrated over the 24‐week study period.

Four board certified dermatologists (J.Y. P., D. J. I., H. D. J., and H. J. K.), each with over 20 years of filler experience, participated in this NLF study. They were convened to discuss their impressions of the PDLLA‐based product and to compare it with the PLLA based product. Although both yielded similar overall outcomes, the panel agreed that NFL effacement appeared more pronounce with the PDLLA based product immediately after injection, following absorption of the carrier solution/gel (sterile water, lidocaine, and carboxymethyl cellulose (only in injectable PLLA)). Unlike PLLA, for which the NLF often returns to its pretreatment state within days after the first injection, PDLLA produces immediate plumping, likely due to the combined effects of non‐cross linked HA and the porous scaffold structure of PDLLA [15]. However, the perception of this early effect was not quantitatively supported reflected in the WSRS or GAIS scores.

Treatment with injectable PLA potentially elicits a cellular cascade of events (subclinical inflammation) leading to collagen formation, in which the magnitude of response is dependent on the type and volume of the PLA used [27, 28]. PLLA is known to stimulate pro‐inflammatory cytokine production in M1 macrophages [29]. It also induces M2 macrophage polarization which in turn stimulates collagen synthesis by upregulating TGF‐β expression in senescent fibroblasts [30]. On the other hand, PDLLA activates fibroblasts by reducing both macrophage and adipose‐derived stem cell (ASC) senescence [31]. PDLLA increases M2 macrophage expression of NRF2, which stimulates ASC proliferation and secretion of TGF‐β, leading to increased collagen synthesis [31]. Since soft tissue thickening is gradual, starting 4–8 weeks after administration, a practice of three injections, spaced sufficiently apart is recommended as with PLLA [32]. This approach minimizes the risk of overcorrection and reduces the risk of adverse events including development of nodules (anomalous accumulation of PLA, usual onset time 1–2 months after injection) or granuloma (exaggerated inflammatory reaction of the host, onset time between 6 and 24 months after PLA injection). The experts felt more resistance on the PLLA side while performing subsequent injections which we assume is the result of a stronger inflammation induced by the dense and spiky PLLA microparticles.

The PDLLA based product has a gel like consistency and all agreed that it was easy to administer. In contrast, PLLA injections require careful pressure control, as the polygonal shape of PLLA microparticles can clog the cannula [33]. PDLLA also has shorter reconstitution time, which is advantageous when procedures need to be performed on short notice. Notably, newer PLLA formulations now permit immediate preparation [34] and have been approved in several markets.

Since PDLLA microspheres are smooth, spongiform and degrade faster than the solid PLLA particles, it is theoretically less likely to cause nodules or granulomas but is not free from the complication [35]. Although cannula delivery by experts allowed the PDLLA based product and PLLA to be well‐tolerated and safe, we experienced a case that needed intervention. This particular patient had a mild NLF and while the first PDLLA based product injection (2 mL of Juvelook Volume) was well tolerated, the second injection with the same amount of filler material caused moderate swelling and tenderness due to the excess localization of HA. Our experience re‐emphasizes the importance of an underfill and careful touch‐up with both the PDLLA based product and PLLA.

The main limitation of this study is its relatively short follow‐up period, which precludes thorough evaluation of the long‐term complications of biostimulators. Additionally, the exclusive use of subjective assessment tools (GAIS and WSRS) limits the robustness of efficacy interpretation, while variations in lighting and camera angles in the photographs may introduce bias.

Our positive clinical experience, in combination with the results presented here support the use of the PDLLA based product for the NLFs. The biocompatible, biodegradable, bio‐stimulatory, and long‐lasting nature of PDLLA is promising but requires further studies incorporating more male subjects, individuals with different Fitzpatrick prototypes and longer follow‐ups to generalize our claim.

## Funding

This work was supported by Vaim Co., Ltd. (Okcheon, Korea).

## Conflicts of Interest

The authors declare no conflicts of interest.

## Data Availability

The data that support the findings of this study are available from the upon reasonable request.
